# ARRB1 downregulates acetaminophen-induced hepatoxicity through binding to p-eIF2α to inhibit ER stress signaling

**DOI:** 10.1007/s10565-024-09842-z

**Published:** 2024-01-22

**Authors:** Yujun Luo, Yiming Lei, Haoxiong Zhou, Yan Chen, Huiling Liu, Jie Jiang, Chengfang Xu, Bin Wu

**Affiliations:** 1 Department of Gastroenterology, The Third Affiliated Hospital of Sun Yat-Sen University, Guangzhou, Guangdong People’s Republic of China; 2 Guangdong Provincial Key Laboratory of Liver Disease Research, Guangzhou, Guangdong People’s Republic of China; 3 Department of Gynecology and Obstetrics, The Third Affiliated Hospital of Sun Yat-Sen University, Guangzhou, Guangdong People’s Republic of China

**Keywords:** Cellular communication, G protein–coupled receptors, Drug-induced liver injury, Apoptosis

## Abstract

Acetaminophen (APAP) stands as the predominant contributor to drug-induced liver injury (DILI), and limited options are available. β-Arrestin1 (ARRB1) is involved in numerous liver diseases. However, the role of ARRB1 in APAP-induced liver injury remained uncertain. Wild-type (WT) and *ARRB1* knockout (KO) mice were injected with APAP and sacrificed at the indicated times. The histological changes, inflammation, endoplasmic reticulum (ER) stress, and apoptosis were then evaluated. Hepatic cell lines AML-12 and primary hepatocytes were used for in vitro analyses. Systemic *ARRB1*-KO mice were susceptible to APAP-induced hepatotoxicity, as indicated by larger areas of centrilobular necrosis area and higher levels of ALT, AST, and inflammation level. Moreover, *ARRB1*-KO mice exhibited increased ER stress (indicated by phosphorylated α subunit of eukaryotic initiation factor 2 (p-eIF2α)-activating transcription factor 4 (ATF4)-CCAAT-enhancer-binding protein homologous protein (CHOP)) and apoptosis (indicated by cleaved caspase 3). Further rescue experiments demonstrated that the induction of apoptosis was partially mediated by ER stress. Overexpression of *ARRB1* alleviated APAP-induced ER stress and apoptosis. Moreover, co-IP analysis revealed that ARRB1 directly bound to p-eIF2α and eIF2α. ARRB1 protected against APAP-induced hepatoxicity through targeting ER stress and apoptosis. ARRB1 is a prospective target for treating APAP-induced DILI.

**Graphical Abstract:**

ARRB1 mitigates APAP-induced hepatotoxicity through regulating ER stress (p-eIF2α-ATF4-CHOP) and apoptosis (p-JNK and cleaved caspase 3) via binding to p-eIF-2α

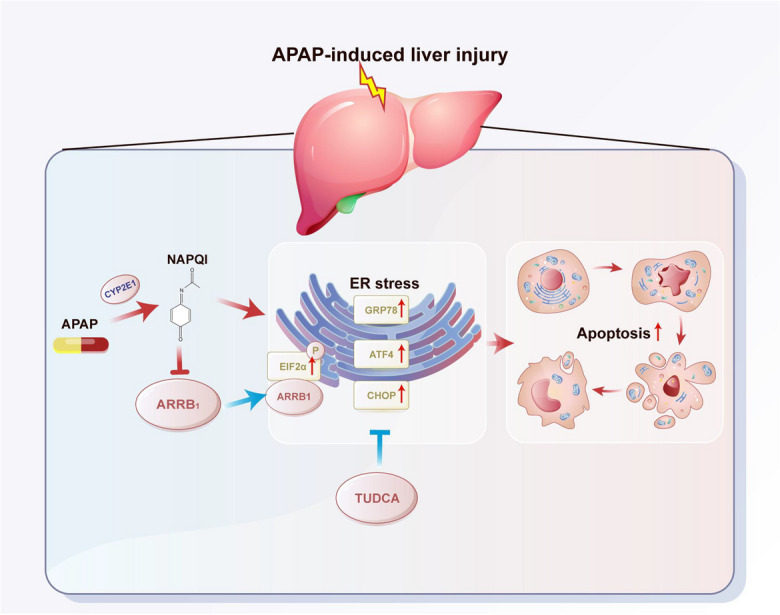

## Introduction

Drug-induced liver injury (DILI) represents the most prevalent cause of acute liver injury (ALI) (Ostapowicz et al. 2002). In the context of the COVID-19 pandemic, acetaminophen (APAP) is one of the most frequently used pain relievers and antipyretic drugs worldwide. While generally considered safe, APAP can lead to intrinsic drug-induced liver injury (DILI) either in a dose-dependent manner or within therapeutic doses due to individual variations, which is characterized by hemorrhagic centrilobular necrosis and elevated plasma transaminase levels (Ramachandran and Jaeschke 2019). Hepatotoxicity ensues from the accumulation of its reactive metabolite, N-acetyl-p-benzoquinone imine (NAPQI), which promptly depletes glutathione (GSH), disrupts mitochondrial functions, triggers endoplasmic reticulum stress, and instigates cell death of liver cells (Yan et al. 2018).

The endoplasmic reticulum (ER) is a crucial organelle responsible for regulating protein folding and modification. Emerging evidence has demonstrated ER stress plays a pivotal role in APAP-induced liver injury (Li et al. 2020; Tak et al. 2022; Torres et al. 2019; Uzi et al. 2013; Ye et al. 2022). Dotan Uzi found that deficiency of CCAAT-enhancer-binding protein homologous protein (*CHOP*) alleviated APAP-induced liver injury in mice (Uzi et al. 2013). Kyu found that genetic ablation of X-box binding protein 1 (*XBP1*) reduced c-Jun N-terminal kinase (JNK) activation and protected mice from APAP-induced liver injury (Ye et al. 2022). A study by Annelies showed that the combined application of the ER stress inhibitor tauroursodeoxycholic acid and N-acetylcysteine exerted a better therapeutic effect on APAP-induced liver injury than conventional therapy (Paridaens et al. 2017). These findings suggested that the modulation of ER stress signaling could be an approach for treating APAP-induced liver injury and the precise mechanism requires further study.

Beta-arrestin-1 (ARRB1) is a ubiquitous multifunctional adaptor protein that serves as a scaffold and adaptor to modulate cellular signaling pathways such as proliferation, apoptosis, and differentiation (Kang et al. 2005; Xiao et al. 2007). Many studies have suggested that ARRB1 plays an important part in the onset and progression of various diseases such as sepsis, cerebral ischemia, and asthma (Sharma et al. 2014; Wang et al. 2014; Pera et al. 2015). According to our previous studies, *ARRB1* deficiency alleviated acute pancreatitis, lipopolysaccharide-induced acute liver injury, radiation-induced intestinal injury, and liver fibrosis (Tao et al. 2019; Lei et al. 2021; Liu et al. 2019; Tan et al. 2015). Furthermore, ARRB1 is closely associated with ER stress. It is reported that overexpression of *ARRB1* inhibits endoplasmic reticulum (ER) stress induced by lipopolysaccharide (LPS) in liver macrophages, while *ARRB1* deficiency exacerbates radiation-induced ER stress in intestinal stem cells (Lei et al. 2021; Liu et al. 2019). Additionally, ARRB1 suppresses endoplasmic reticulum (ER) stress and the PUMA pathway, thereby reducing apoptosis in gastric mucosa associated with portal hypertension gastropathy (Tan et al. 2015). Taken together, we suspected that ARRB1 plays a pivotal role in APAP-induced liver injury possibly via ER stress signaling.

Despite numerous studies emphasizing the significance of ARRB1 in liver diseases, there is a lack of research focusing on the role of ARRB1 in APAP-induced liver injury. Currently, N-acetylcysteine (NAC) stands as the sole pharmacological therapy for APAP-induced liver injury, but its efficacy is contingent upon the timing of administration relative to intoxication (Paridaens et al. 2017). Additionally, long-term NAC use might inhibit liver regeneration, as shown in an experimental model (Paridaens et al. 2017). Taken together, the development of potentially effective therapies for treating APAP overdose is of the utmost importance. The result of our study showed that *ARRB1* deficiency exacerbated APAP-induced liver injury indicated by severer injury area, unfolded protein response, and apoptosis. Further inhibition of ER stress partially reversed the severe injury induced by *ARRB1* knockout. Overall, these findings indicate that ARRB1 provided partial protection against APAP-induced liver injury, potentially through its involvement in endoplasmic reticulum (ER) stress modulation, and ARRB1 represents a promising therapy for addressing APAP-induced liver injury.

## Method

### Animal model and treatment

All animal experiments conducted in this study received approval from the Institutional Animal Care and Use Committee at the Third Affiliated Hospital of Sun Yat-Sen University (ethics committee approval number: IACUC-F3-23–0615). *ARRB1*-KO mice were generated as previously described (Lei et al. 2021). During the experiment, mice were housed with sufficient food and water under a pathogen-free condition (12-h light/dark cycle).

For in vivo APAP-injury study, male mice (23 ± 2 g, 8 weeks old) were intraperitoneally injected with 400 mg/kg APAP (MCE cat: HY-66005), after being fasting for 12 h. Pair-fed control mice were intraperitoneally injected with a sample volume of PBS.

For inhibition of ER stress, mice were administered TUDCA (100 mg/kg body weight; MCE cat: HY-19696A) in PBS (vehicle solution) by intraperitoneal injection 2 h prior the intraperitoneal administration of 400 mg/kg APAP (Du et al. 2022; Sun et al. 2020). The mice were euthanized 12 h later using carbon dioxide inhalation as the humane method of sacrifice. For survival analysis, 21 mice were intraperitoneally injected with 750 mg/kg APAP.

### Blood and liver tissue collection

Blood was collected using the eyeball blood collection method. Briefly, we compressed the mouse eyeballs to enhance their protrusion, and then, we quickly extracted the eyeballs using forceps while simultaneously collecting blood into an EP tube. The blood was incubated at 4 °C overnight and then centrifuged at 3000 r/min. The supernatants were collected for further use.

Liver tissues were collected via a midline incision. After washing with PBS, the whole liver was separated into several parts for further use. Tissue homogenization was performed using Bead Ruptor 12 (OMINI international) following the manufacturer’s instructions.

### Serological analysis

Serum alanine aminotransferase (ALT) and aspartate aminotransferase (AST) levels were measured as previously described (Kehua Biology, Shanghai, China, 370,457, 371,368) (Lei et al. 2021).

### Inflammation factor assessment

Serum IL-1β, IL-6, and TNF-α levels as well as hepatic level were measured by ELISA kit (Servicebio, GEM0003, GEM0001, GEM0004) following the instruction.

### Primary hepatocyte isolation and treatment

As described previously, hepatocytes were isolated from WT and *ARRB1*-KO mice by 2-step collagenase perfusion. In brief, the mice were anesthetized and subjected to a 10-min perfusion with a calcium-free buffer solution. Subsequently, perfusion with 0.05% type IV collagenase (Sigma-Aldrich, St. Louis, MO, USA, cat: G5138) was performed in the portal vein. After being minced, mice livers were filtered through 70-μm filters. Afterwards, liver cells were separated by performing two rounds of centrifugation at a force of 50 g for a duration of 2 min. The isolated hepatocytes were cultured in collagen-coated plates in preparation for subsequent experiments. Cells were then incubated with or without 10 mM APAP for 12 h.

### Cell culture and treatment

AML-12 cells were seeded in Dulbecco’s Modified Eagle Medium/Nutrient Mixture F-12 (DMEM-F12) (Gibco BRL, Rockville, MD, USA, cat: C11330500BT) supplemented with 10% heat-inactivated fetal bovine serum (FBS, cat: FSP500) and 1% ITS. Cells were incubated with 5% CO_2_ at 37 ℃. For APAP treatment, AML-12 cells were incubated in a cultured medium containing 10 mM APAP for 24 h, with or without 1 mM TUDCA for the indicated times.

### Cell viability assessment

Cell viability was evaluated according to the provided instructions (Dojindo Cell Counting Kit-8, cat: CK04). AML-12 cells were seeded in a 96-well culture (1 × 10^4^ cells/well). Following APAP administration, cells were incubated in a cultured medium containing 10 μL of CCK-8 for 1 h at 37 °C. Cell viability was measured as optical density (OD) using 450 nm using Multiskan Spectrum (Thermo Fisher).

### Plasmid transfection and interference with ARRB1

*ARRB1* plasmid was bought from Umine Biotechnology Co., Ltd (Guangzhou). Plasmid transfection was performed based on the instructions of jetPRIME (Polyplus, France, cat: 101,000,046).

According to jetPRIME instructions, *ARRB1* siRNA was transfected into cells for silencing. The sequence was 5′-CCUUGAGGCAUCACUGGAUAAdTdT-3′ (tsingke, China).

### RNA extraction and real-time PCR for gene expression

RNA was isolated from liver tissues or cells using Trizol regent for quantitative real-time PCR (Promega, Madison, WI, USA). According to the manufacturer’s instructions, reverse transcription was performed using Reverse Transcription Kit (TOYOBO, Japan, cat: FSQ-201). SYBR Green (Invitrogen, USA) was used to assess the levels of mRNA for relevant genes with a Mini Opticon Real-Time PCR System (Bio-Rad, Hercules, CA, USA). As a normalized control, we used β-actin as a basis for determining gene expression levels. Primer sequences are listed in Table 1.

**Table 1:** Primer sequences

Mouse	Primer sequences	NCBI accession number
*ARRB1*	5′-CCGAGGACAAGAAGCCACTGA-3′ (sense)	109689
	5′-AGAGTGACTGAGCATGGAAGGT-3′ (antisense)	
*β-actin*	5′-TCTCCTTCATGCGTTGCTT-3′ (antisense)5′-GGCTGTATTCCCCTCCATCG-3′ (sense)	11461
*ARRB2*	5′-AGTCGAGCCCTAACTGCAAG-3′ (sense)	216869
	5′-ACGAACACTTTCCGGTCCTTC-3′ (anti-sense)	
*GRP78*	5′-GCCGAGGAGGAGGACAAGAA-3′ (sense)	14828
	5′-ACACACCGACGCAGGAATAG-3′ (antisense)	
*CHOP*	5′-CCCTCGCTCTCCAGATTCC-3′ (sense)	13198
	5′-TCTCCTTCATGCGTTGCTT-3′ (antisense)	

### Immunohistochemistry

Mice were euthanized and sacrificed on 12 h after APAP injection. Paraffinized sections were incubated with anti-CHOP (Cell Signaling Technology, 2895) at 4 ℃ overnight. The secondary antibodies were used according to the Envision kit (DAKO, Carpinteria, CA, cat: GK500710).

The necrosis index (scoring system) for liver injury is established by Suzuki, which has 5 grades (0, no necrosis; 1, single cell necrosis; 2, necrosis < 30%; 3, 30% < necrosis < 60%; 4, necrosis > 60%) (Suzuki et al. 1993).

### Immunofluorescence

Mice were euthanized and sacrificed on 12 h after APAP injection. Paraffinized sections were incubated with anti-p-eIF2α (Affinity # AF3087) used for the primary antibody. For secondary antibody, anti-rabbit Alexa Fluor 594 (Cell Signaling Technology Cat# 8889, RRID: AB_2716249) or anti-rabbit Alexa Fluor 488 (Abcam Cat# ab150077, RRID: AB_2630356) was used. The nuclear staining was performed using DAPI. A fluorescent microscope (OLYMPUS BX43) was used to photograph.

For immunocytochemistry, cells were incubated with anti-eIF2α (ZenBio Cat# 201,137) and anti-ARRB1(Abcam Cat# 32,099) at 4 ℃ overnight. Anti-rabbit Alexa Fluor 594 (Invitrogen Cat# A11037) and anti-mouse Alexa Fluor 488 (Invitrogen Cat# A11001) were used as secondary antibodies. The nuclear staining was performed using DAPI.

### Protein extraction and Western blot

To extract proteins from tissue samples, 20 mg tissue was homogenized in 200 µL RIPA buffer for 40 s and lysed at 4 °C for 2 h. After centrifugation for 20 min (12,000 × g), the supernatant was collected for protein concentration analysis by the BCA method or boiled with a loading buffer for further use.

To extract proteins from cell samples, cells were lysed in RIPA buffer (100 µL/well). After centrifugation for 20 min (12,000 × g), the supernatant was collected for protein concentration analysis by the BCA method or boiled with a loading buffer for further use.

Antibodies against ARRB1 (Abcam, ab32099), ARRB2 (Sigma-Aldrich, SAB2500117), Cyp2e1 (Proteintech, 19,937–1-AP), Glucose Regulated Protein 78 (GRP78) (Cell Signaling Technology, 3177), p-eIF2α (Abclonal, AP0692), ATF4 (Proteintech, 10,835–1-AP), eIF2α (Cell Signaling Technology, 5324), CHOP (Cell Signaling Technology, 2895), cleaved caspase 3 (Affinity, AF7022), anti-JNK1 + JNK2 + JNK3 (phospho T183 + T183 + T221) (Abcam, ab124956), SAPK/JNK (Cell Signaling Technology, 9252), and GAPDH (Sigma-Aldrich, A5441), anti-Bax antibody (Abcam, ab32503), Bcl2 monoclonal antibody (Proteintech, 68,103–1-Ig), β-actin (Cell Signaling Technology, 3700), and α-tubulin (Ray antibody biotech RM2007) were used as primary antibodies.

Signals were evaluated by ECL chemiluminescence (Thermo Fisher Scientific, Waltham, MA, USA). Normalized controls included GAPDH, β-actin, and α-Tubulin. Data represents the ratio to the control group after normalization.

### Liver GSH, SOD, MDA assay

GSH levels were assessed in liver tissues (20–50 mg) based on the instructions of commercially available kits (Boxbio, Beijing, cat: AKPR008M).

Superoxide dismutase (SOD) activity and malonaldehyde (MDA) levels were assessed by commercially available kits (Boxbio, Beijing, China, cat: AKAO001M, AKFA013M).

### TUNEL staining

Apoptosis of samples obtained from mouse liver tissue or cells was analyzed using the deoxynucleotidyl transferase dUTP nick-end labeling (TUNEL) assay (Servicebio, cat: G1502). Nuclear staining was performed using DAPI. Each group includes at least 3 mice.

### Flow cytometry

To detect cell death, the Annexin V-FITC/PI Apoptosis Detection Kit (BD Bioscience, US, cat: 556,547) was used to quantify the apoptosis of AML-12 24 h after incubation with 10 mM APAP. Briefly, (1 × 10^5^ cells/well) AML-12 cells were seeded in 6-well plates. After incubation with APAP and intervention at the indicated time, cells were digested by trypsin, suspended, and incubated with reagents from the detection Kit. Flow cytometry was used to quantify the cells.

### Co-immunoprecipitation (CO-IP) assay

Proteins from AML-12 were lysed in Pierce™ IP Lysis Buffer after experimental treatments. Lysates were incubated with protein A/G magnetic beads (MCE, cat: HY-K0203) at 4 °C overnight and then incubated with anti-ARRB1 (Abcam, ab32099), anti-eIF2α (ZenBio Cat# 201,137), anti-p-eIF2α (Abclonal, cat: AP0745) antibody, or IgG antibodies (Beyotime, A7016; Beyotime, A7028) overnight at 4 °C. The magnetic beads were washed with PBST and boiled in a loading buffer at 70 °C for 10 min. The supernatants were collected and subjected to Western blot.

### Statistical analyses

All data were expressed as the mean ± SD. Data analysis was performed using GraphPad Prism version 8.0. A two-tailed Student’s *t* test or one-way analysis of variance (ANOVA) was employed to determine statistical significance. A significance level of *P* < 0.05 was used to determine statistical significance. Each experiment was independently replicated at least three times, and consistent results were obtained.

## Result

### ARRB1 is downregulated in APAP-induced hepatoxicity

**Fig. 1:**
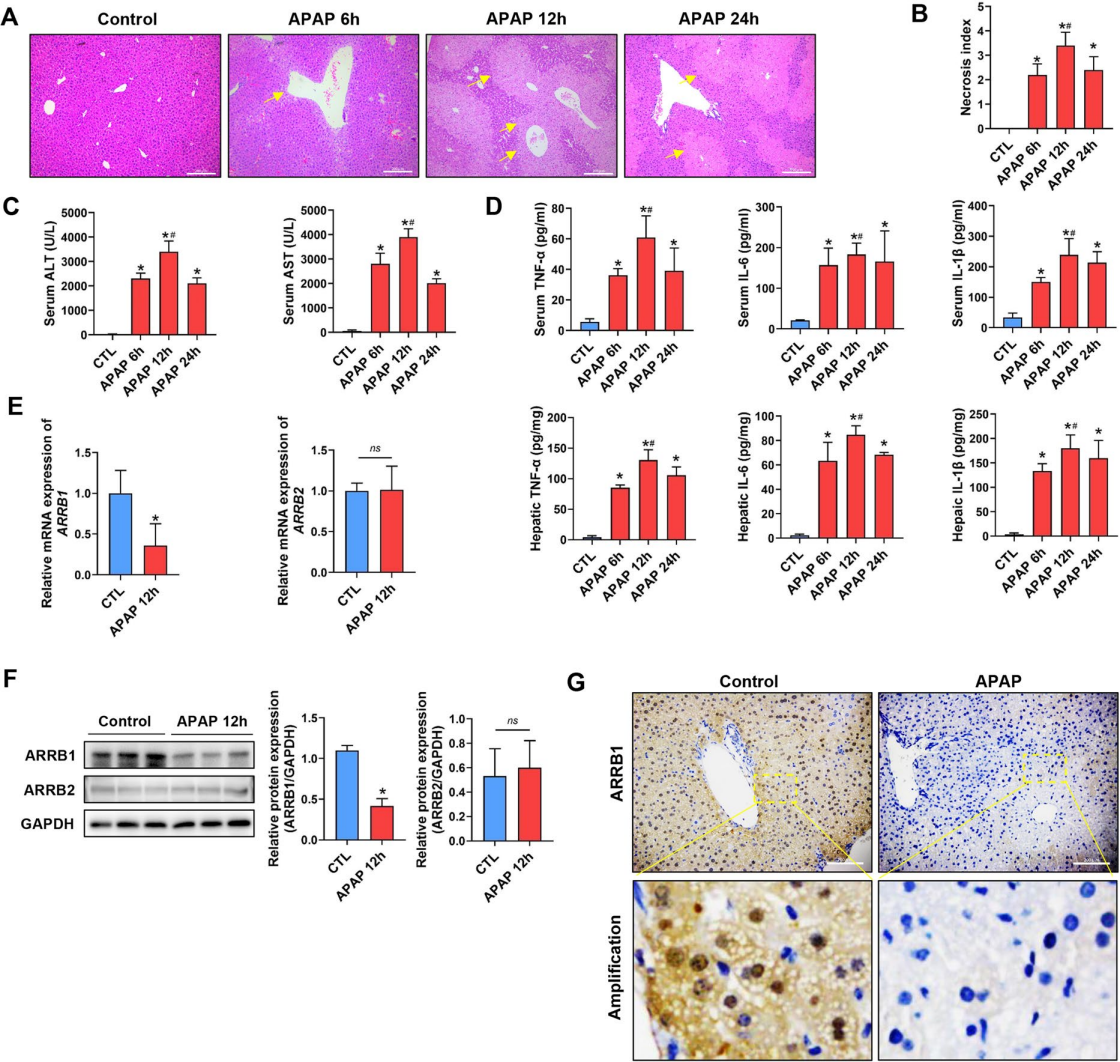
ARRB1 is downregulated in APAP-induced liver injury. **A** Histological analysis of liver tissue by H&E staining. Scale bar: 200 μm. **B** Necrosis index is calculated by Suzuki. Data represents 3 ~ 5 animals. **C** Serum ALT and AST levels in WT mouse at different times after injection. *n* = 3. **D** Serum and hepatic IL-1β, TNF-α, and IL-6 levels in WT mouse at different times after injection. *n* = 3 ~ 5. **E** *ARRB1* and *ARRB2* mRNA levels in vehicle- and APAP-induced WT mouse liver tissues were analyzed by real-time PCR. All values are the mean ± SD (*n* = 3 in each group). **F** Western blotting analysis of ARRB1 and ARRB2 in vehicle- and APAP-induced WT mouse liver tissues at 12 h with GAPDH as the internal control. Data represent the mean ± SD (*n* = 3). **G** ARRB1 staining of livers in vehicle- and APAP-induced WT (scale bar: 200 μm). *P < 0.05 compared with the WT group; #*P* < 0.05 compared with APAP 6 h or APAP 24 h by Student’s *t* test

To establish APAP-induced liver injury, 400 mg/kg APAP was intraperitoneally injected into mice. HE staining showed that APAP mice exerted the most injury area at 12 h with mild recovery at 24 h (Fig. 1A, B). Consistent with the above findings, the level of serum levels of AST and ALT as well as TNF-α, IL-1β, and IL-6 peaked at 12 h suggesting that the optimal time to investigate injury is 12 h (Fig. 1C, D). We then evaluated whether ARRB1 or ARRB2 was involved in APAP-induced hepatoxicity. The PCR, Western blot, and IHC demonstrated that APAP significantly downregulated ARRB1, while there was a limited change in ARRB2 expression (Fig. 1E, F, G). Taken together, these findings indicated the potential involvement of ARRB1 in APAP-induced hepatoxicity.

### Systemic ARRB1 deficiency exacerbates APAP-induced hepatotoxicity in mice

**Fig. 2:**
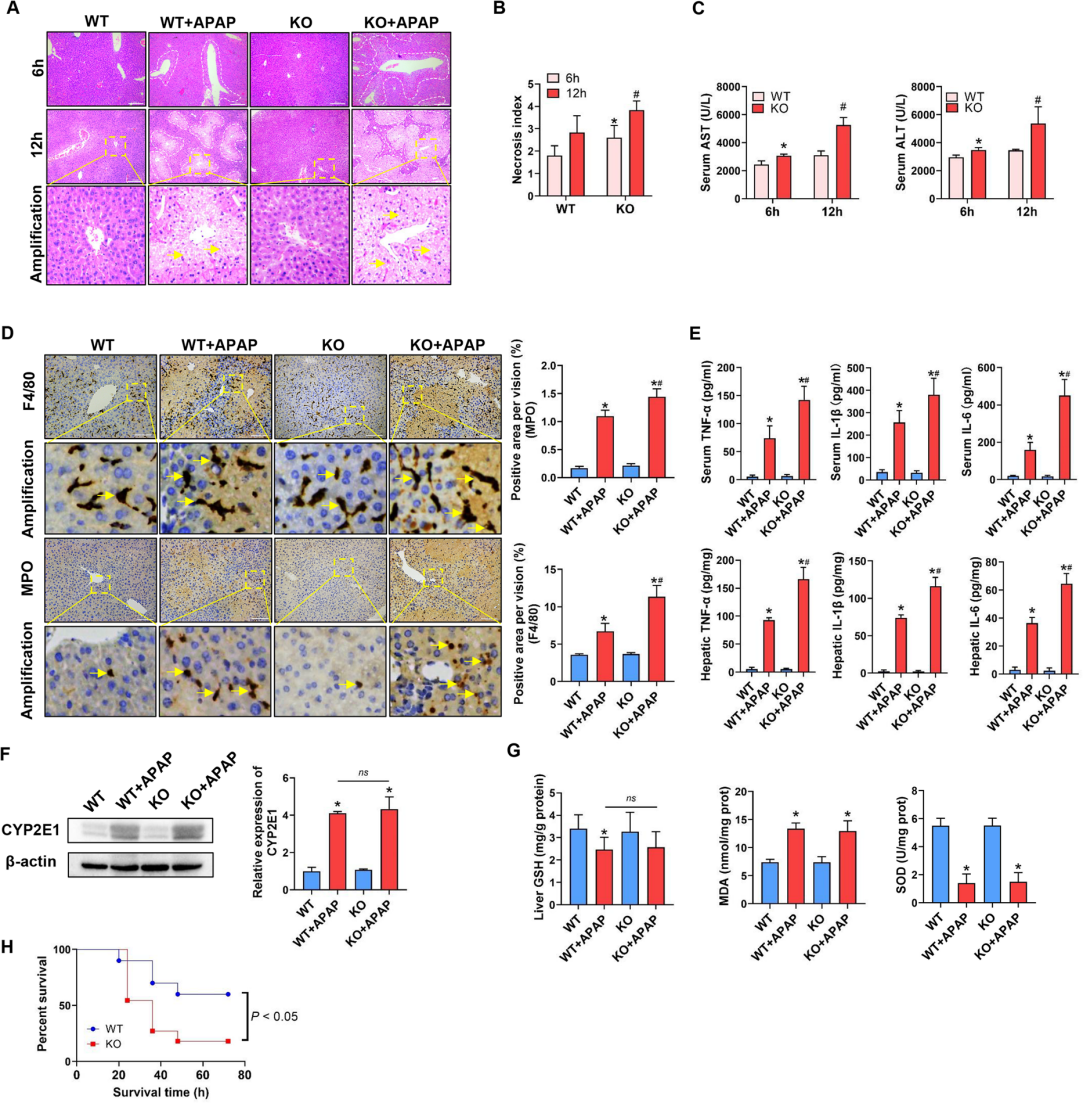
Systemic *ARRB1* deficiency exacerbates APAP-induced hepatotoxicity in mice. **A** Histological analysis of liver tissue by H&E staining. Scale bar: 200 μm. **B** Necrosis index is calculated by Suzuki. Data represents 3 ~ 5 animals. *P < 0.05 WT + APAP 6 h vs *ARRB1*-KO + APAP 6 h. #*P* < 0.05 WT + APAP 12 h vs *ARRB1*-KO + APAP 12 h. **C** Serum ALT and AST levels in WT mouse at different times after injection. *n* = 3 ~ 5. *P* < 0.05 WT + APAP 6 h vs *ARRB1*-KO + APAP 6 h. #*P* < 0.05 WT + APAP 12 h vs *ARRB1*-KO + APAP 12 h. **D** Intrahepatic staining of F4/80 (macrophages) and MPO (neutrophils). Arrows indicate positive staining. Scale bar: 200 μm. Each group has at least 3 mice. *P < 0.05 compared with the WT group; #*P* < 0.05 compared with WT + APAP group by Student’s *t* test. **E** Hepatic and serum IL-1β, TNF-α, and IL-6 levels in mouse at 12 h after injection. *n* = 3 ~ 5. *P < 0.05 compared with the WT group; #*P* < 0.05 compared with WT + APAP by Student’s *t* test. **F** Western blotting analysis of Cyp2e1 in vehicle- and APAP-induced WT mouse liver tissues at 12 h with GAPDH as the internal control. Data represent the mean ± SD (*n* = 3). *P < 0.05 compared with the WT group. **G** Liver GSH, MDA, and SOD in the indicated group. *n* = 3. *P < 0.05 compared with the WT group. **H** Cumulative survival for WT or *ARRB1*-KO mice exposed to APAP (750 mg/kg) was analyzed using the Kaplan–Meier method. *P* values were determined by log-rank testing

To investigate the possible role of ARRB1 in vivo, WT mice or *ARRB1*-KO mice were subjected to APAP (400 mg/kg, 12 h) or PBS. According to HE staining, *ARRB1*-KO mice exhibited severer injury indicated by more injury area 6 h and 12 h after injection compared with WT mice (Fig. 2A, B, 6h: *P* = 0.03, 12 h:* P* = 0.003). Consistent with these findings, *ARRB1*-KO mice have a higher level of AST, ALT, TNF-α, IL-1β, and IL-6 and more inflammatory infiltrates as indicated by higher expression of F4/80 and MPO than WT mice after APAP injection (Fig. 2C, D, E, AST: *P* = 0.048; ALT:* P* = 0.036; TNF-α, IL-1β, IL-6:* P* = 0.016, *P* = 0.015, 0.0001; F4/80: *P* = 0.012; MPO: *P* = 0.026). To further explore the impact of *ARRB1* deficiency on metabolism, we then evaluated the protein level of Cyp2e1. The results showed that *ARRB1* deficiency had a limited effect on Cyp2e1 expression (Fig. 2F, *P* = 0.58). Additionally, there was no significant difference in GSH between the WT + APAP and *ARRB1*-KO + APAP groups (Fig. 2G, *P* = 0.85). Moreover, when injected with APAP (750 mg/kg), *ARRB1*-KO mice had a poorer survival time than WT mice (Fig. 2H, *P* = 0.046). Collectively, this evidence demonstrated that *ARRB1* deficiency exacerbates the hepatotoxicity induced by APAP and that ARRB1 protects against APAP-induced liver injury without affecting drug metabolism.

### ARRB1 relieves ER stress during APAP-induced liver injury

**Fig. 3:**
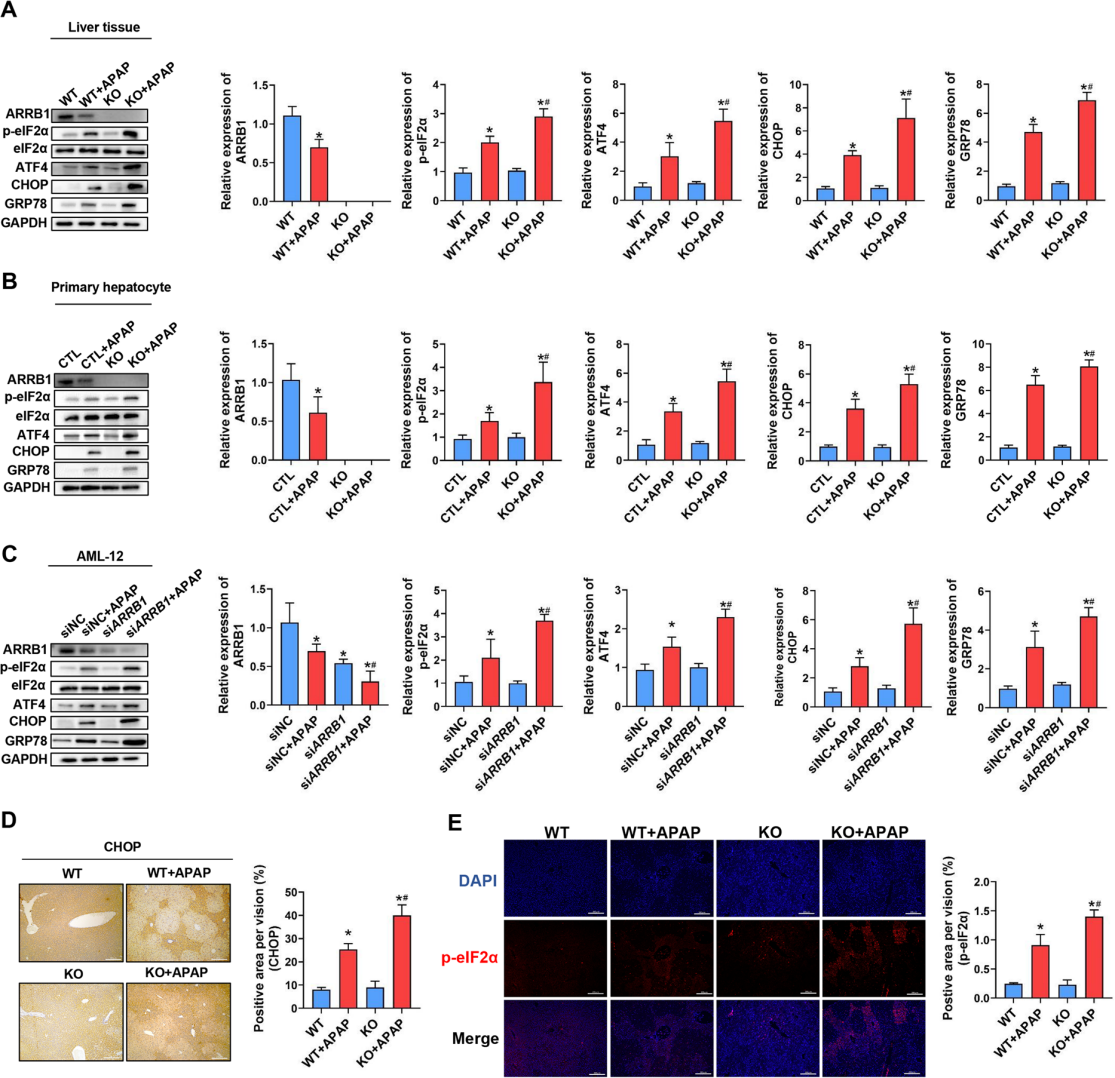
ARRB1 relieves ER stress in APAP-induced hepatotoxicity. **A** Western blot analysis of liver tissue from WT or *ARRB1*-KO mice with or without injection of APAP. Each group has at least 3 mice. *P < 0.05 compared with the WT group; #*P* < 0.05 compared with WT + APAP group by Student’s *t* test. **B** Western blot analysis of primary hepatocyte from WT or *ARRB1*-KO mice with or without APAP. Data are shown as the mean ± SD. *P < 0.05 compared with the control group; #*P* < 0.05 compared with control + APAP group by Student’s *t* test. **C** Western blot analysis of AML-12 with or without incubation of APAP for 24 h. *P < 0.05 compared with the control group; #*P* < 0.05 compared with control + APAP group by Student’s *t* test. Data are shown as the mean ± SD. **D** Intrahepatic staining of CHOP staining. Scale bar: 200 μm. Each group has at least 3 mice. *P < 0.05 compared with the WT group; #*P* < 0.05 compared with WT + APAP group by Student’s *t* test. **E** Immunofluorescence of p-eIF2α in liver tissue. Scale bar: 200 μm. Each group has at least 3 mice. *P < 0.05 compared with the WT group; #*P* < 0.05 compared with WT + APAP group by Student’s *t* test

Previous studies have underscored the crucial role of ER stress in acetaminophen (APAP)-induced liver injury. We then investigated whether *ARRB1* deficiency influenced ER stress signaling. A Western blot of liver tissue was performed. As shown by Fig. 3A, ER stress markers p-eIF2α, ATF4, CHOP, and GRP78 were upregulated in WT after APAP treatment, while the markers above were higher in the *ARRB1*-KO + APAP group. To further verify our hypothesis, primary hepatocytes from liver tissue were then evaluated. Consistent with the findings described above, *ARRB1* knockout exacerbated the ER stress signaling induced by APAP as indicated by Western blot (Fig. 3B). To further verify the role of ARRB1 on ER stress, murine hepatocyte cell line AML-12 was incubated with 10 mM APAP for 24 h. The expression levels of ER stress markers p-eIF2α, ATF4, CHOP, and GRP78 were higher in the *ARRB1* knockdown + APAP group compared to the control + APAP group (Fig. 3C).

As shown by IHC and IF, there were no differences in the baseline of CHOP and p-eIF2α expression between WT mice and *ARRB1*-KO mice after overnight fasting (Fig. 3D, E, eIF2α: *P* = 0.79, CHOP: *P* = 0.57). CHOP and p-eIF2α expression was significantly increased after APAP injection in WT mice, while the trend was more obvious in *ARRB1*-KO mice, suggesting that ARRB1 might modulate the ER stress signaling in APAP-induced liver injury (Fig. 3D, E, p-eIF2α: *P* = 0.017, CHOP: *P* = 0.0083).

Collectively, these findings suggest that the deficiency of *ARRB1* exacerbates ER stress signaling in both in vivo and in vitro models of acetaminophen (APAP)-induced hepatotoxicity.

### ARRB1 mitigates APAP-induced apoptosis

**Fig. 4:**
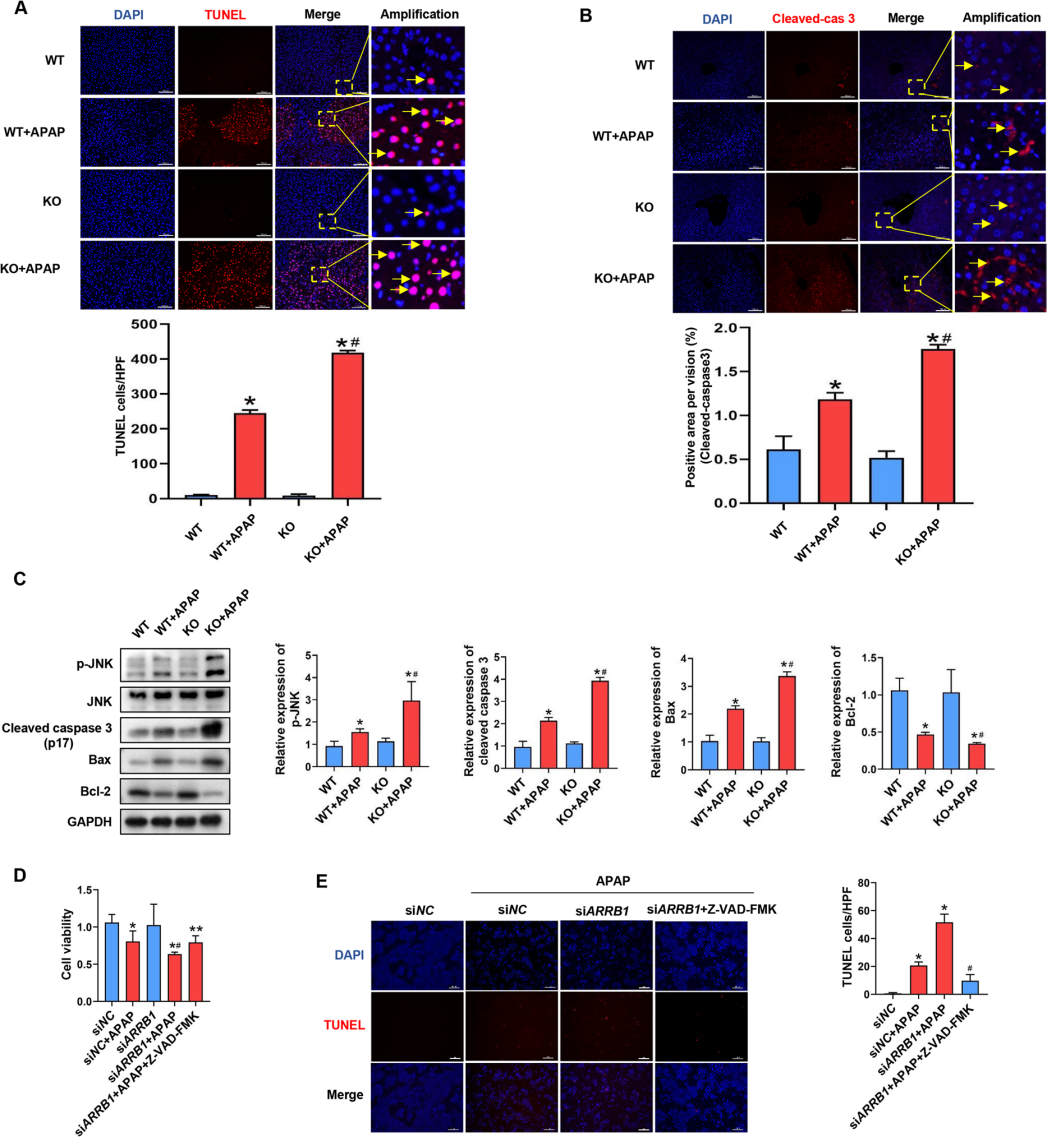
ARRB1 mitigates APAP-induced apoptosis. **A** Apoptosis was assayed by TUNEL staining at 12 h after APAP injection. Each group has at least 3 mice. Arrowheads indicate TUNEL-positive cells. *P < 0.05 compared with the WT group; #*P* < 0.05 compared with WT + APAP group by Student’s *t* test. **B** Immunofluorescence staining with cleaved caspase 3. Liver samples were collected and stained 12 h after injection. Arrowheads indicate a positive signal. Scale bar: 200 μm. Each group has at least 3 mice, *P < 0.05 compared with the WT group; #*P* < 0.05 compared with WT + APAP group by Student’s *t* test. **C** Western blot of liver tissue from WT or *ARRB1*-KO mice with or without injection of APAP. Data represents at least 3 samples. *P < 0.05 compared with the WT group; #*P* < 0.05 compared with WT + APAP group by Student’s *t* test. **D** Cell viability of AML-12 incubated with APAP or Z-VAD-FMK is detected by CCK-8. Data represent mean ± SD of three independent experiments. *P < 0.05 compared with the control group; #*P* < 0.05 compared with control + APAP group by Student’s *t* test; **P < 0.05 compared with the *siARRB1* + APAP group. **E** Immunofluorescence staining of AML-12 cells with TUNEL 24 h after APAP incubation. Scale bar: 100 μm. Data represent mean ± SD of three independent experiments. *P < 0.05 compared with the si*NC* group; #*P* < 0.05 compared with si*NC* + APAP group

Apoptosis has been reported to occur in APAP-induced hepatoxicity and we further investigated the role of ARRB1 in APAP-induced apoptosis. As shown in Fig. 4A, TUNEL staining indicated a strong apoptosis signal in WT mice injected with APAP compared to WT mice, suggesting that APAP induced apoptosis in mice (Fig. 4A). Moreover, *ARRB1*-KO mice had more TUNEL-positive cells than WT mice after APAP injection. Consistent with the TUNEL staining, *ARRB1*-KO mice injected with APAP had significantly higher expression of cleaved caspase 3, Bax, and phospho-SAPK/JNK (p-JNK) and lower expression of Bcl-2 than WT mice injected with APAP as indicated by immunofluorescence and Western blot (Fig. 4B and C). To assess the impact of ARRB1 in APAP-induced apoptosis, AML-12 were incubated with 10 mM APAP for 24 h and cellular viability was evaluated by CCK-8. As shown by Fig. 4D, *ARRB1* knockdown aggravated the APAP-induced cell death which could be partially alleviated by a pan-caspase inhibitor (Z-VAD-FMK). Further TUNEL staining revealed that Z-VAD-FMK could strongly reduce the number of TUNEL-positive cells in *ARRB1* knockdown AML-12 incubated with APAP (Fig. 4E). In conclusion, ARRB1 mitigated APAP-induced apoptosis.

## Blocking ER stress partially reverses APAP-induced apoptosis in *ARRB1*-KO mice

As preceding data demonstrated that *ARRB1* deficiency intensified ER stress and facilitated apoptosis during APAP-induced hepatoxicity, we further wanted to know whether ARRB1 modulated apoptosis through ER stress signaling.

**Fig. 5:**
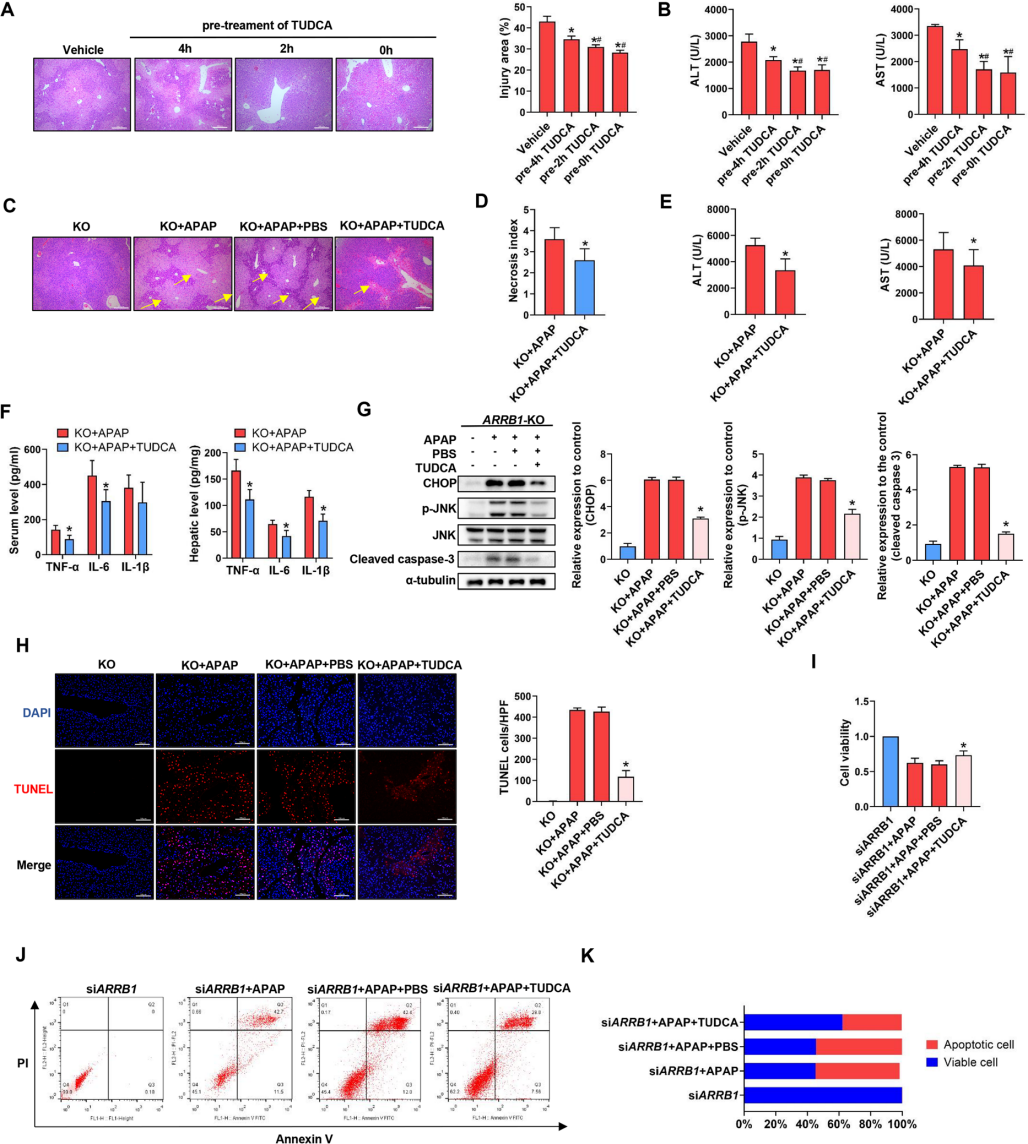
ER stress partially reverses *ARRB1* deficiency–mediated liver injury. **A** Histological analysis of liver tissue from mice injected with APAP and TUCDA at different times by H&E staining. Scale bar: 200 μm. *n* = 3 ~ 5. *P < 0.05 compared with the vehicle group. #*P* < 0.05 compared with the pre-4 h TUDCA group. **B** Serum ALT and AST levels. *P < 0.05 compared with the vehicle group. #*P* < 0.05 compared with the pre-4 h TUDCA group. **C** Histological analysis of liver tissue by H&E staining. Scale bar: 200 μm. *n* = 3 ~ 5. *P < 0.05 compared with the *ARRB1*-KO + APAP group. **D** Necrosis index is calculated by Suzuki. Data represents 3 ~ 5 animals. *P < 0.05 compared with the *ARRB1*-KO + APAP group. **E** Serum ALT and AST levels in *ARRB1*-KO mice + APAP or *ARRB1*-KO mice + APAP + TUDCA. *n* = 3 ~ 5. *P < 0.05 compared with the *ARRB1*-KO + APAP group. **F** Serum and hepatic IL-1β, TNF-α, and IL-6 levels in *ARRB1*-KO mice + APAP or *ARRB1*-KO mice + APAP + TUDCA. *n* = 3 ~ 5. *P < 0.05 compared with the *ARRB1*-KO + APAP group. **G** Western blot of liver tissue from WT or *ARRB1*-KO mice with APAP injection or TUDCA injection. Data represent mean ± SD of three independent experiments. *P < 0.05 compared with the *ARRB1*-KO + APAP group. **H** TUNEL staining of WT or *ARRB1*-KO mice with APAP injection or TUDCA injection. *P < 0.05 compared with the *ARRB1*-KO + APAP group. **I** Cell viability of AML-12 incubated with APAP or Z-VAD-FMK is detected by CCK-8. Data represent mean ± SD of three independent experiments. *P < 0.05 compared with the *ARRB1*-KO + APAP group. **J** Apoptosis of AML-12 was evaluated by flow cytometry after PI/Annexin V staining 24 h after APAP incubation. The left upper quadrant contains necrotic cells (%); the upper right quadrant contains late apoptotic cells (%); the lower left quadrant contains live cells (%); and the lower right quadrant contains early apoptotic cells (%). **K** The percentage of total apoptotic cells and viable cells under each condition are shown

To screen for the best time for tauroursodeoxycholic acid (TUDCA) treatment, mice injected with APAP were injected with TUDCA at different times. As shown by HE staining and the level of AST and ALT, pretreating mice with TUDCA 0 h or 2 h before the experiment exerts the best protective effect compared to 4 h, while there is no significant difference between 0 and 2 h **(**Fig. 5A, B**)**. These findings suggest that pretreating mice 2 h before the experiment is the optimal time for TUDCA treatment.

*ARRB1*-KO mice were then subjected to APAP injection with PBS or TUDCA. As expected, treatment with TUDCA significantly decreases the injury area, and the level of serum ALT, AST, and inflammatory factors compared to the PBS group, suggesting the blockage of ER stress partially reversed the *ARRB1* deficiency–induced phenotypes (Fig. 5C, D, E, F). Western blot of liver tissues revealed that TUDCA partially inhibited the expression of CHOP, p-JNK, and cleaved caspase 3 in the *ARRB1*-KO + APAP group, while Tunnel staining showed that the increase of Tunnel positive cells was partially revered by TUDCA (Fig. 5G, H). To further demonstrate the ER stress signaling and apoptosis, AML-12 were incubated with APAP 10 mM for 24 h. As shown by CCK-8, the cell viability was partially rescued in the *ARRB1*-KO + APAP + TUDCA group (Fig. 5I). Consistent with the CCK-8 analysis, flow cytometry revealed that TUDCA partially reversed the apoptosis in the *siARRB1* + APAP group (Fig. 5J, K). Taken together, these findings indicated that ARRB1 modulated apoptosis in APAP-induced liver injury through ER stress signaling.

## Overexpression of *ARRB1* alleviates APAP-induced ER stress and apoptosis

**Fig. 6:**
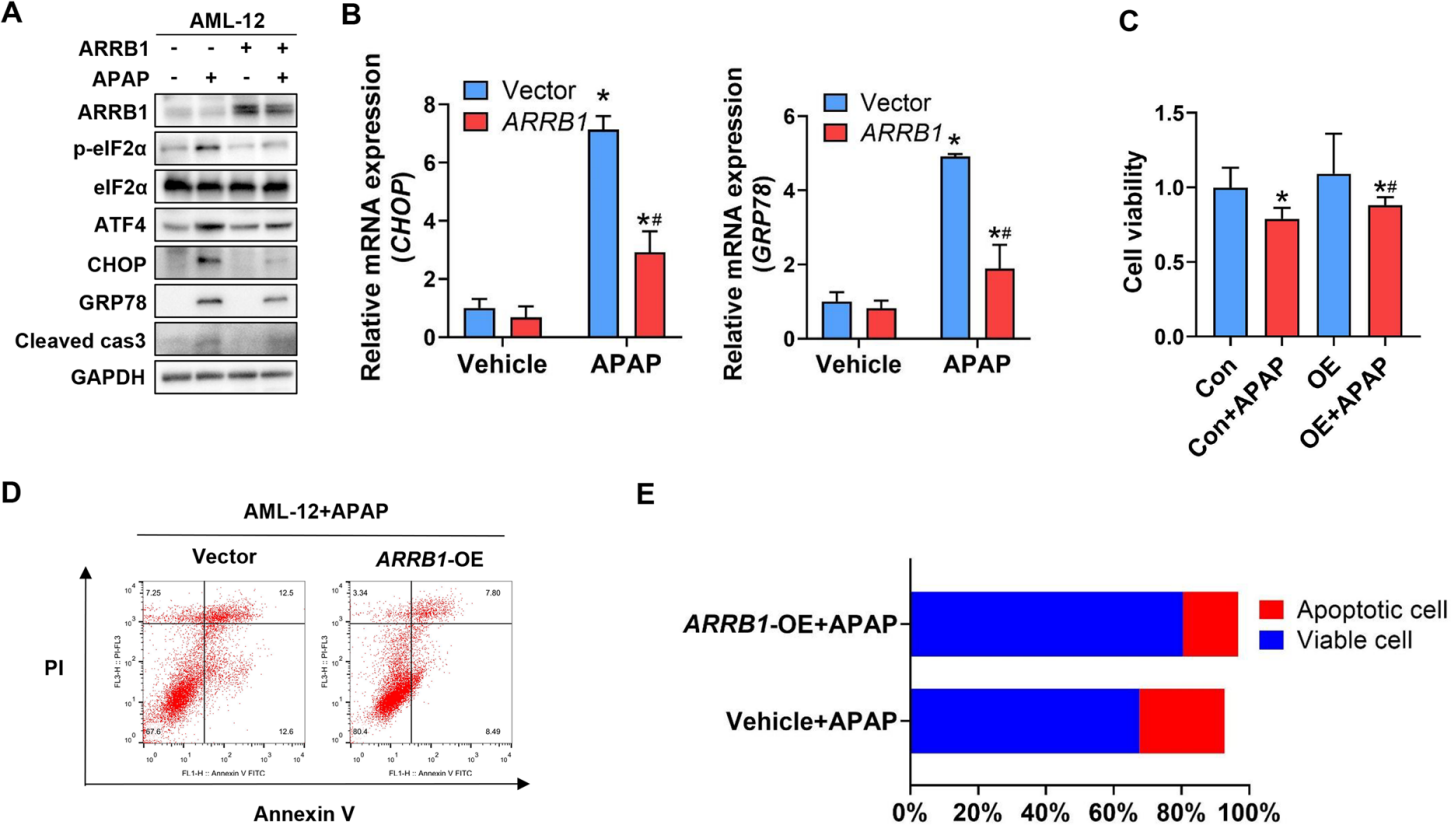
Overexpression of *ARRB1* alleviates APAP-induced ER stress and apoptosis. **A** Western blot analysis of AML-12 with or without incubation of APAP for 24 h. **B** CHOP and GRP78 mRNA levels in vehicle- and APAP-treat AML-12 were analyzed by real-time PCR. All values represent 3 independent experiments. *P < 0.05 compared with the vehicle group; #*P* < 0.05 compared with OE + APAP group by Student’s *t* test. **C** Cell viability of AML-12 incubated with or without APAP is detected by CCK-8. Data represent mean ± SD of three independent experiments. *P < 0.05 compared with the vehicle group; #*P* < 0.05 compared with OE + APAP group by Student’s *t* test. **D** Apoptosis of AML-12 was evaluated by flow cytometry after PI/Annexin V staining 24 h after APAP incubation. The left upper quadrant contains necrotic cells (%); the upper right quadrant contains late apoptotic cells (%); the lower left quadrant contains live cells (%); and the lower right quadrant contains early apoptotic cells (%). **E** The percentage of total apoptotic cells and viable cells under each condition are shown. OE overexpression

To investigate the role of ARRB1 in APAP-induced hepatotoxicity, we then overexpressed *ARRB1* in AML-12. We treated AML-12-vector and AML-12-overexpressing *ARRB1* with or without APAP. Western blot analysis showed that the markers of ER stress and apoptosis were partially suppressed in the *ARRB1*-OE + APAP group compared with the vector + APAP group (Fig. 6A). The qPCR also showed that the mRNA levels of *CHOP* and *GRP78* in the *ARRB1*-OE + APAP group were lower than those in the vector + APAP group (Fig. 6B, *P* = 0.001). These findings suggested that overexpression of *ARRB1* suppressed ER stress during APAP overdose.

To explore the impact of ARRB1 on APAP-induced cell death, we then performed CCK-8 and flow cytometry on AML-12. The CCK-8 analysis showed that overexpression of *ARRB1* significantly increased the cell viability in the *ARRB1*-OE + APAP group compared with the vector + APAP group (Fig. 6C, *P* = 0.03). Consistent with the above findings, flow cytometry showed that the ratio of apoptosis was reduced in the *ARRB1*-OE + APAP group compared with the vector + APAP group (Fig. 6D, E), indicating that overexpression of *ARRB1* alleviated the apoptosis induced by APAP in liver cells. Taken together, overexpression of *ARRB1* suppressed the ER stress and apoptosis induced by APAP.

## ARRB1 directly binds to p-eIF2α during APAP overdose

**Fig. 7:**
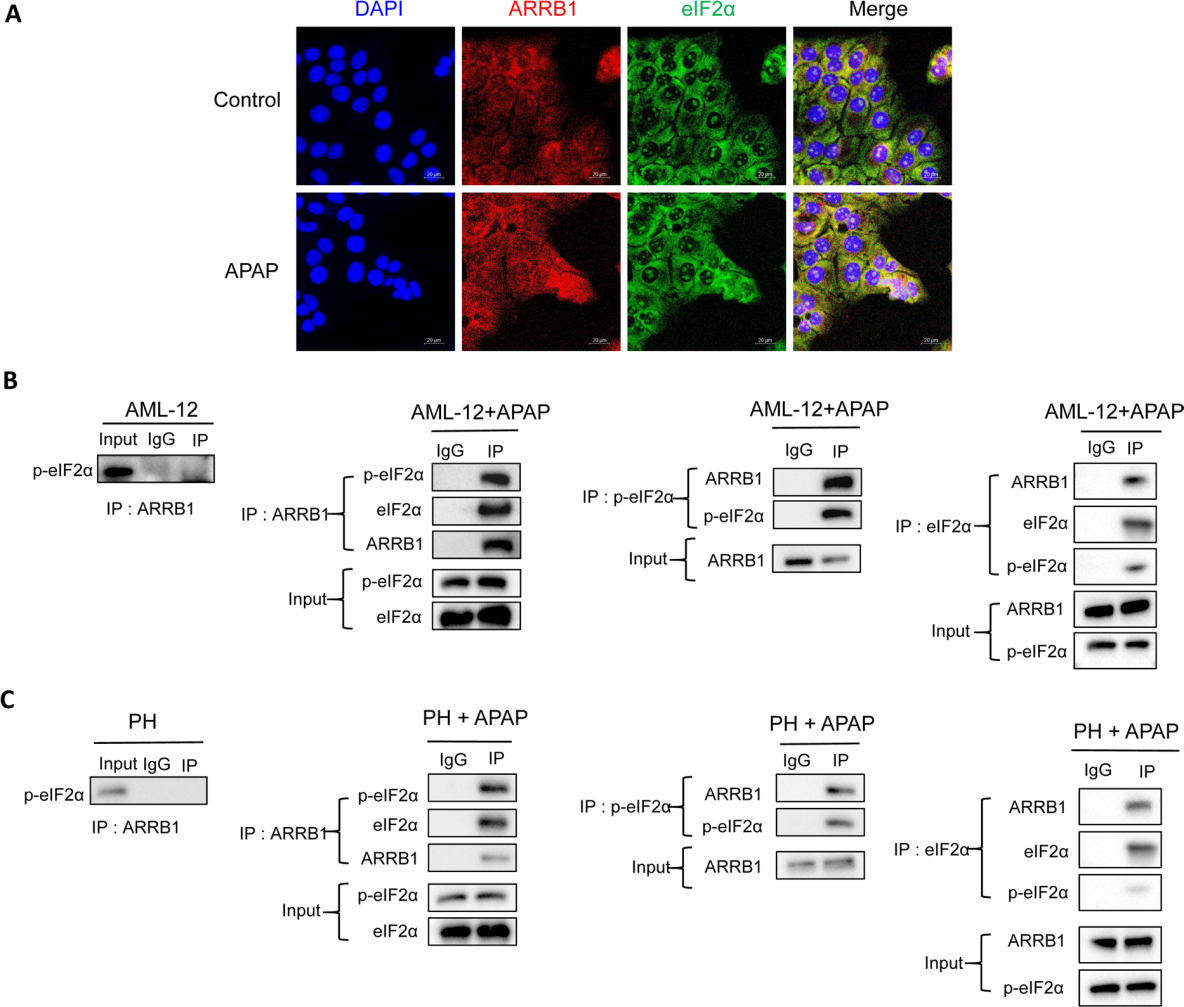
ARRB1 directly binds to p-eIF2α in APAP-induced liver injury. **A** Double immunostaining of ARRB1 and eIF2α in AML-12. Red = ARRB1, green = eIF2α. **B** The cellular lysates of AML-12 were subjected to immunoprecipitation with anti-ARRB1, anti-eIF2α, anti-p-eIF2α, or IgG antibody. Co-immunoprecipitated endogenous p-eIF2α, eIF2α, and ARRB1 were detected with anti-p-eIF2α, anti-eIF2α, and anti-ARRB1 antibodies as indicated. **C** Primary hepatocytes were isolated from mice with or without APAP injection. The cellular lysates of primary hepatocytes were subjected to immunoprecipitation with anti-ARRB1, anti-p-eIF2α, or IgG antibody. Co-immunoprecipitated endogenous p-eIF2α, eIF2α, and ARRB1 were detected with an anti-p-eIF2α or eIF2α, ARRB1 antibody as indicated. PH primary hepatocyte

As our findings have demonstrated that ARRB1 regulated ER stress signaling in APAP-induced liver injury, we further wanted to know how ARRB1 functions with eIF2α. As shown in Fig. 7A, ARRB1 colocalized with eIF2α in AML-12 cells, suggesting there should be an interaction between these 2 proteins (Fig. 7A).

To further explore the interaction between ARRB1 and eIF2α, we then performed a co-immunoprecipitation (Co-IP) assay. In normal conditions, the Co-IP experiment reveals no interaction between ARRB1 and p-eIF2α in AML-12. Then, after incubation with APAP, an interaction between ARRB1 and p-eIF2α or eIF2α was confirmed by co-IP (Fig. 7B). Consistent with these findings, the co-IP revealed no evidence of interaction between ARRB1 and p-eIF2α in primary hepatocytes isolated from WT mice, while the co-IP indicated a strong interaction between ARRB1 and p-eIF2α (Fig. 7C). These findings suggested that ARRB1 might modulate ER stress by directly binding to p-eIF2α.

## Discussion

Our study confirmed the hypothesis that ARRB1 alleviates acetaminophen-induced hepatoxicity and showed that (1) *ARRB1* deficiency aggravated the necrosis and inflammation induced by APAP injection. (2) *ARRB1* deficiency exacerbated the ER stress and apoptosis in APAP overdose. (3) Blockage of ER stress mitigated the ARRB1-induced apoptosis. (4) Overexpression of *ARRB1* alleviated ER stress and apoptosis induced by APAP overdose. (5) ARRB1 directly bound to p-eIF2α after APAP treatment (Fig. 8).

There are some significant findings of our work. First, our study is the first study that reveals the protective impact of ARRB1 on APAP-induced hepatoxicity. ARRB1, a member of the arrestin family, was initially recognized as a versatile adaptor protein with the primary function of negatively regulating the biological process of GPCRs, such as desensitization and internalization. Although previous studies have reported the protective role of ARRB1 in many liver diseases, such as non-alcoholic steatohepatitis, liver ischemia/reperfusion injury, and lipopolysaccharide-induced acute liver injury, there is no report about the role of ARRB1 in APAP-induced liver injury (Xu et al. 2020; Zhang et al. 2020; Lei et al. 2021). In our study, ARRB1 expression was significantly downregulated in mice that received APAP injection, and systematic deficiency of *ARRB1* exacerbated the APAP-induced hepatoxicity as indicated by more injury area and inflammation infiltration. Thus, ARRB1 plays a protective role in APAP-induced hepatoxicity.

Second, we found that ARRB1 regulated ER stress signaling and apoptosis in APAP overdose. ER stress and apoptosis are two important biological processes that regulate APAP-induced hepatoxicity, while suppression of these processes decreases APAP-induced hepatotoxicity (Ramachandran and Jaeschke 2019). In our previous study, Lei discovered that ARRB1 suppressed LPS-induced hepatic macrophage activation via ER stress signaling (Lei et al. 2021). Tan reported that ARRB1 protected against the ER stress/PUMA signaling pathway in hypertensive gastropathy (Tan et al. 2015). These studies suggest that ER stress and apoptosis are downstream of ARRB1. Consistent with these findings, we found that ARRB1 alleviated APAP-induced liver injury through regulating ER stress-p-eIF2α-ATF4-CHOP signaling. As the majority of cell types of the liver are hepatocytes, we then isolated primary hepatocytes from mouse livers and observed the same activation of ER stress-p-eIF2α-ATF4-CHOP signaling in liver cells, indicating that the beneficial effect of ARRB1 on APAP overdose mainly occurred in hepatocytes. JNK, one of the members of the mitogen-activated protein kinase (MAPK) superfamily, regulates various biological functions. Sustained activation of JNK leads to necrosis and apoptosis (Leppa and Bohmann 1999). In numerous studies, pharmacological JNK inhibition by SP610025 strongly inhibited apoptosis in APAP overdose, suggesting JNK is one of the key members in regulating apoptosis (Gunawan et al. 2006; Shi et al. 2019). Additionally, some studies showed that activation of ER stress induces p-JNK (Ye et al. 2022). In our study, we detected enhanced activation of apoptosis, marked by increased levels of p-JNK and cleaved caspase 3, in the *ARRB1*-KO + APAP group in comparison to the WT + APAP group. Further rescue experiments confirmed that the apoptosis and JNK activation were partially mediated by ER stress signaling. Moreover, overexpression of *ARRB1* partially rescued the ER stress and apoptosis induced by APAP.

eIF2α is one of the key proteins in ER stress signaling. The phosphorylation of eIF2α, which is induced by numerous cellular stresses, causes inhibition of global translation in order to maintain homeostasis. However, the phosphorylation of eIF2α also preferentially translates integrated stress response (ISR) genes, especially ATF4 and CHOP. The unregulated ISR triggers several downstream events such as apoptosis, inflammation, or necrosis (Wek 2018). In our study, we observed that deficiency of *ARRB1* inhibits the eIF2α-ATF4-CHOP axis, thus enhancing apoptosis and exacerbating liver injury.

Third, we found that there is a direct interaction between ARRB1 and eIF2α or p-eIF2α. Although previous studies confirmed the strong correlation between ARRB1 and ER stress, no studies have reported the underlying mechanism. In our study, we found that there is no direct interaction between ARRB1 and p-eIF2α under normal conditions. The interaction between ARRB1 and p-eIF2α or eIF2α became more prominent after APAP treatment, suggesting that ARRB1 might regulate ER stress via directly binding to p-eIF2α.

Glutathione (GSH) plays a crucial role in detoxifying acetaminophen (APAP) by converting N-acetyl-p-benzoquinone imine (NAPQI) into a non-toxic form, thereby preventing APAP-induced liver injury. Following a toxic dose of APAP, approximately 90% of liver GSH is depleted and APAP-protein adducts are formed, resulting in hepatic toxicity. In our study, we discovered that there is no difference in GSH level between the WT + APAP and *ARRB1*-KO + APAP groups suggesting that ARRB1 has a limited effect on the detoxification and the metabolism of APAP. The protective role of ARRB1 is primarily achieved through modulation of signaling pathway.

There are some limitations and future prospects in our studies. First, although we determined that ARRB1 directly binds to p-eIF2α and eIF2α, the mechanism by which ARRB1 regulates eIF2α phosphorylation remains unclear. Second, the clinical application of ARRB1 is a challenging and extensive journey. Tanaka revealed that high-purity omega-3 polyunsaturated fatty acids (n-3 PUFAs) alleviated NASH patients as indicated by liver fat, liver enzymes, or markers of inflammation, while a study from Kenneth showed that ARRB1 variants may influence the response to antidepressant drugs in depressed patients (Chappell et al. 2022; Tanaka et al. 2008). As ARRB1 alleviates APAP-induced hepatoxicity, the development of ARRB1-specific activators is urgent.

## Conclusion

All in all, our study suggests that ARRB1 relieved APAP-induced hepatoxicity through targeting ER stress and apoptosis signaling. More studies are needed to develop an ARRB1 activator to treat APAP-induced liver injury.

## Data Availability

The datasets used and/or analyzed during the current study are available from the corresponding author on reasonable request.

## Abbreviations

APAP: Acetaminophen
DILI: Drug-induced liver injury
GPCRs: G protein–coupled receptors
WT: Wild-type
KO: Knockout
ER: Endoplasmic reticulum
